# Positive effects of audit and feedback on antimicrobial use in hospitalized patients limited to audited patients

**DOI:** 10.1017/ash.2024.37

**Published:** 2024-04-16

**Authors:** Elske M. Engel-Dettmers, Nashwan Al Naiemi, Hero E. Dijkema, Annemarie L.M.A. Braakman-Jansen, Lisette J.E.W.C. van Gemert-Pijnen, Bhanu Sinha

**Affiliations:** 1 Faculty of Behavioral, Management and Social Sciences, Psychology, Health and Technology, University of Twente, Enschede, The Netherlands; 2 Department of Clinical Pharmacy, ZGT, Almelo and Hengelo, The Netherlands; 3 Department of Medical Microbiology, Labmicta, Hengelo, The Netherlands; 4 Department of Urology, ZGT, Almelo and Hengelo, The Netherlands; 5 Department of Medical Microbiology and Infection Prevention, University Medical Center Groningen, Groningen, The Netherlands

## Abstract

**Objective:**

Audit and feedback is an antimicrobial stewardship (AMS) strategy, with the potential to also optimize antimicrobial use in non-audited patients. This study aimed to determine whether audit and feedback reduce antimicrobial use in both audited and non-audited patients.

**Design:**

Before-after trial with a 1-year intervention period and 2.5-year historical cohort.

**Setting:**

750-bed community hospital in the Netherlands.

**Patients:**

All patients admitted to the urology wards during the 3.5-year study period were observed. Patients were classified as using antimicrobials if any antimicrobial was used for therapeutic reasons. Patients using antimicrobials prophylactically were excluded from measurements.

**Intervention:**

The AMS team provided audit and feedback on antimicrobial use for patients using antimicrobials for 2 days. Retrospectively, antimicrobial use and length of stay (LOS) were compared with the historical cohort.

**Results:**

Audits modified antimicrobial treatment in 52.8% of the cases. De-escalating, stopping, and switching from intravenous to oral treatment accounted for 72% of these modifications. Compared to patients from the cohort, who also used antimicrobials for 2 days, antimicrobial use decreased from 14.21 DDD/patient (95% CI, 13.08–15.34) to 11.45 DDD/patient (95% CI, 8.26–14.64; *P* = .047) for audited patients. Furthermore, mean LOS decreased from 7.42 days (95% CI, 6.79–8.06) to 6.13 days (95% CI, 5.38–6.89; *P* = .031). However, looking at all patients admitted to the urology wards, the percentage of patients using antimicrobials and total antimicrobial use remained unchanged.

**Conclusions:**

Audit and feedback reduce antimicrobial use and LOS, but only for audited patients. Positive effects are not automatically transferred to patients for whom no audits have been performed.

## Introduction

Appropriate treatment of infections and judicious use of antimicrobials are important strategies to prevent the development of antimicrobial resistance.^1–4^ Antimicrobial stewardship (AMS) is an organizational approach for optimizing the use of antimicrobials and thus preserving their effectiveness in the future.^5^ Although antimicrobial use and the burden of antimicrobial resistance are relatively low in the Netherlands, an AMS program has been mandatory for all Dutch hospitals since 2014.^4,6–8^ In countries with low resistance rates, intensive monitoring of antimicrobial use and resistance development is intended to keep these rates low, and to ensure that any increase in antimicrobial resistance is detected in time to take effective action.^9^ The paradox between low resistance rates on the one hand and the call for intensive monitoring on the other may explain the low priority that some Dutch hospitals give to AMS. As a result, their AMS teams struggle with a lack of manpower and support to properly implement AMS programs. Every year, the Dutch Working Party on Antibiotic Policy (Dutch acronym: SWAB) conducts a survey that shows that 33% of the respondents have no budget for AMS activities.^9^ Because of this capacity problem, AMS teams need to focus on implementing AMS activities with the greatest clinical impact.^10,11^

AMS programs involve various activities. The Dutch AMS guideline distinguishes between AMS objectives and AMS strategies. Objectives are the activities carried out at the patient level, such as taking cultures before starting treatment and following treatment guidelines. The strategies refer to the ways in which these objectives should be achieved, such as by antimicrobial restriction, education, or decision support through audit and feedback. The Dutch AMS guideline recommends which AMS objectives should be pursued but does not recommend which strategy should be used to achieve these objectives.^12^

A Cochrane review by Davey *et al* showed that all studied AMS strategies had a positive impact on antimicrobial use. By their negative nature, however, restricting measures, raise concern about postponement of treatment and loss of confidence between professionals. Including persuasive or empowering measures in the strategies increases the positive effect of AMS strategies.^13^ Audit and feedback is one of the widely accepted persuasive activities with internationally proven effects on antimicrobial use and length of stay (LOS).^14,15^

Dik *et al* showed that their audit and feedback strategy not only reduced antimicrobial use in audited patients but also in non-audited patients admitted to the same hospital ward.^16^ Given the lack of resources for one-third of the Dutch AMS teams and the labor-intensity of audit and feedback activities, which is an obstacle that AMS teams worldwide are struggling with,^9,11^ the transcending effect seen by Dik *et al* offers an opportunity that has not been shown by others and warrants further research in other settings. The aim of this study was to determine whether the audit and feedback strategy reduces antimicrobial use not only in audited patients but also in non-audited patients in a Dutch community hospital.

Because urinary tract infections are among the most common infections with high recurrence rates and increasing antimicrobial resistance, this study was conducted in the hospital’s urology wards.^17,18^

## Methods

### Setting

In this before-after study, with a historical cohort control, the hospital’s AMS team provided audit and feedback on 2 urology wards for every patient using any antimicrobial for 2 days from December 2016 to November 2017. The study took place in a large 750-bed community hospital in the Netherlands, at two different locations (Almelo and Hengelo). Typically, patients are admitted to the urology wards for urinary tract surgery, such as cystectomy, prostatectomy, and transurethral endoscopic procedures, or for urinary retention and urinary tract infections. The AMS team is a multidisciplinary team, consisting of hospital pharmacists, clinical microbiologists, and medical specialists with a focus on infectious diseases. The team reports to the hospital’s antimicrobial committee, which is responsible for the implementation and operation of the AMS program.

### Patients

All patients admitted to the hospital by urologists between June 2014 and November 2017 were observed. Urology admissions were defined as registrations as urology admissions in the hospital information system (HIS: HiX, Chipsoft BV, Amsterdam, The Netherlands) at discharge. Patients were classified as antimicrobial users if an antimicrobial was used for therapeutic reasons, ie, to treat a suspected or confirmed infection. Prophylactic use was defined as antimicrobial use intended to prevent the occurrence or recurrence of an infection. Patients who used antimicrobials prophylactically were manually excluded from the measurements based on the combination of antimicrobial and dosing regimen.

### Intervention

During the intervention period, the hospital pharmacist on the AMS team used the HIS to select patients for daily case-audits on weekdays. When activated, the HIS selected patients admitted to the urology ward who used antimicrobials for at least 2 days. This overview included the patient identification number and prescription details, such as the type of antimicrobial, dosage, and start date (day 0). The AMS-team hospital pharmacist and clinical microbiologist completed this information with clinical chemistry data (C-reactive protein, leukocytes, and serum creatinine) and (preliminary) microbiology diagnostic reports. The clinical microbiologist then called the patient’s bedside physician, and together, they evaluated the prescribed antimicrobial therapy and the available patient-related infection data. The purpose of this evaluation was to reach agreement on the decision about continuation of treatment. During the case audit, the clinical microbiologist invited the bedside physician to ask additional questions or present other patients for evaluation.

Local guidelines for urological infections, based on national and European guidelines, formed the basis for treatment decisions. Agreed-on treatment decisions and time spent on preparation and execution of the case audits were recorded. After the patients were discharged from the hospital, the hospital pharmacist of the AMS team classified the treatment decisions according to the AMS objectives of the Dutch AMS guideline^12^ and checked compliance within 24 hours after the case audit was performed.

### Measures

Therapeutic antimicrobial use was measured in defined daily doses (DDDs), as stated by the WHO, and indexed in 2018.^19^ LOS was measured in full days. The main endpoints were the effect of the audits on antimicrobial use in the whole ward, measured as DDDs per 100 patient days, and the percentage of patients using antimicrobials. To measure the effect of the audits on antimicrobial use and LOS for those patients who actually received an audit, a frequency-based historical control cohort was constructed. The cohort consisted of all patients admitted for urologic care who stayed in the same wards during a 30-month control period prior to the intervention period, and who used antimicrobials for at least 2 days. All demographic and antimicrobial use data were extracted from the HIS using an overview of all antimicrobials administered to the observed patients during the study period. Case report forms were completed for the audited patients to measure the treatment decisions agreed upon during the audits, such as dose adjustments, antimicrobial changes, or discontinuation of the antimicrobial used. Adherence to these decisions and the time spent by the AMS team conducting the case audits were also recorded on the case report forms.

### Statistics

An interrupted time-series analysis was performed to compare the antimicrobial use of all patients admitted to the urology wards during the case-audit period with that of all patients admitted to the urology wards during the 30-month period, prior to the intervention period. Descriptive statistics included unpaired *t*-tests, χ^2^ tests, and Kaplan–Meier survival plots with a log-rank test were applied, as appropriate. The significance threshold was *P* < .05, and the analysis was performed with SPSS version 28 (IBM, SPSS Statistics. Armonk, New York, USA).

### Ethics

The research protocol was reviewed in accordance with Dutch legislation and the guidelines of the local ethics committee (reference K16-53). Because the research data were anonymized and partially aggregated after collection from the HIS, and patient integrity was not compromised, the committee determined that the study was not subject to the Dutch Medical Research Involving Human Subjects Act.^20^

## Results

Our analysis included 5479 patients admitted to the urology wards. Of these patients, 1475 (27%) used antimicrobials for therapeutic reasons. A total of 622 of these antimicrobial users (11% of all admissions) were admitted for 2 days or more and thus received antimicrobials for at least 2 days during admission. The mean age and sex distribution of antimicrobial users were similar in the control and intervention groups (Table 1).

**Table 1.** Patient baseline characteristics

	Control period	Intervention period	*P*-value
All patients (n)	3853	1626	
Male (%)	75.7	79.3	.004^a^
Mean age (yr)	64.8 (±0.5)	64.5 (±0.7)	.438^b^
Antimicrobial use (n)	1043	432	
Male (%)	71.2	73.1	.458^a^
Mean age (yr)	67.2 (±0.9)	66.0 (±1.5)	.181^b^
Antimicrobial use for 2 d (n)	438	184	
Male (%)	70.5	69.6	.807^a^
Mean age (yr)	69.2 (±1.2)	68.4 (±2.1)	.693^b^

Note. Patient characteristics with their *P*-values and 95% confidence intervals in brackets.

a χ^2^ test.

b Mann-Whitney *U*-test.

Of the 432 patients who used antimicrobials during the intervention year, 184 (42.6%) used them for at least 2 days, and 127 (29.4%) were audited. The 57 patients (13.2%) who used antimicrobials for 2 days but were not audited were missed by the AMS team for organizational reasons. The main reason was that the AMS team was available only on weekdays. During the audits, no additional patients were suggested for review by the bedside physician.

**Figure 1.**
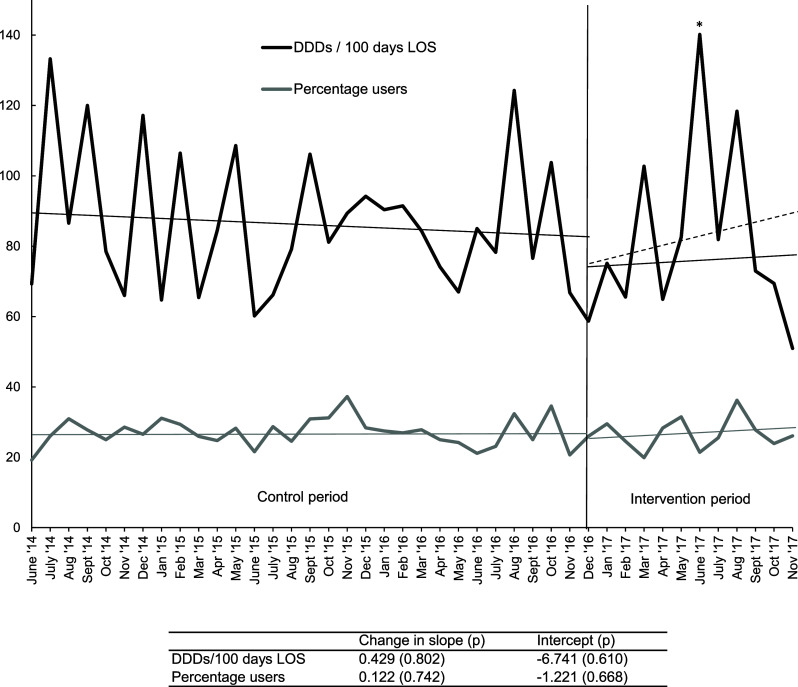
Effects of audit and feedback on antimicrobial use in all admitted patients. Time series of antimicrobial use in all admitted patients as percentage of patients using antimicrobials on the wards and in defined daily doses (DDDs) per 100 patient days, defined as length of stay (LOS). Shown are the 30-month control period before audit and feedback started, and the 12-month intervention period (December 2016 to November 2017).*Note*: *The peak in the month of June is caused by a single patient with a complicated urosepsis, who received extensive antimicrobial treatment. The dotted trend line for the mean DDDs represents the trend including the outlier patient.

The audits performed had no significant effect on antimicrobial use in the total ward. The time-series analysis shows that the intercept for the number of antimicrobial DDDs used per 100 patient days decreased, while the slope increased, both nonsignificant. The same was true for the percentage of patients using antimicrobials (Figure 1).

**Figure 2.**
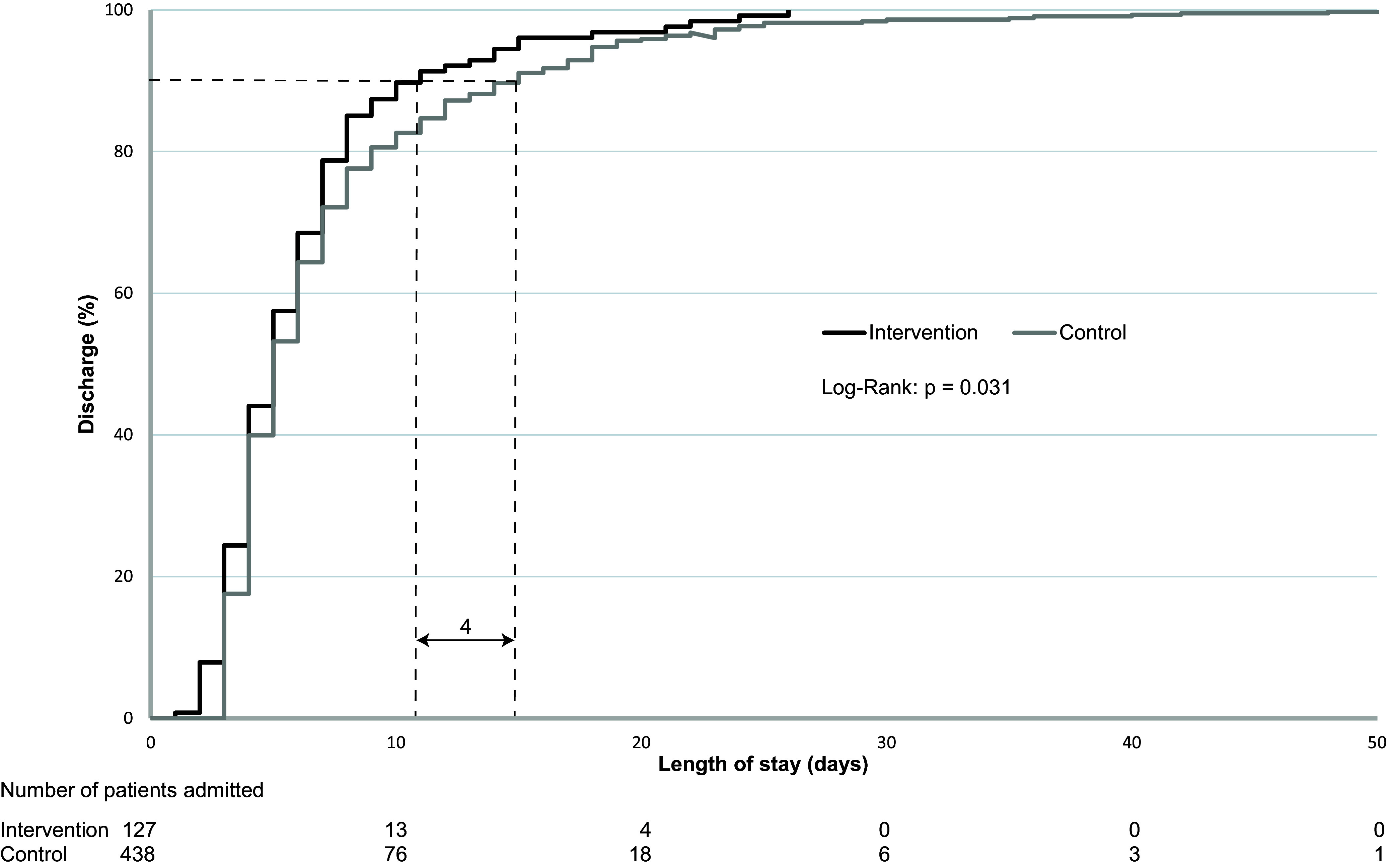
Effect of audit and feedback on length of stay. Kaplan Meier plots of percentage of patients per day of discharge, comparing audited patients (intervention) to the historical control cohort (control). Dotted lines: Ninety percent of audited patients were discharged 4 days earlier. Significance was tested with a log-rank test (Mantel-Cox).

A total of 127 audited patients had a mean age of 66.9 years (95% CI, 64.1–69.7), and 67.7% were male. This was not significantly different from the mean age and sex distribution of patients using antimicrobials for at least 2 days in the historical cohort from Table 1 (*P*-value 0.219 and 0.540 respectively). Compared to those patients from the historical cohort, the case audits significantly reduced LOS and antimicrobial use for the individual patients receiving a case audit. Mean LOS was reduced by 1.29 days (7.42 days; 95% CI, 6.79–8.06 vs 6.13 days; 95% CI, 5.38–6.89, *P* = .031), with the greatest impact on patients with the longest LOS (Figure 2). Antimicrobial consumption decreased by 2.76 DDDs (*P* = .047) per patient in the case-audit group (Table 2).

**Table 2.** Effect of audit and feedback on antimicrobial use

	Control period (n = 438)	Intervention period (n = 127)	Difference (%)	*P*-value
DDDs	14.21 (±1.13)	11.45 (±3.19)	–19.40	.047

Note. DDDs, Defined Daily Doses

Antimicrobial use in mean DDDs per patient, 95% confidence intervals in brackets

*P*-value for difference calculated with unpaired two-tailed *t*-test

**Figure 3.**
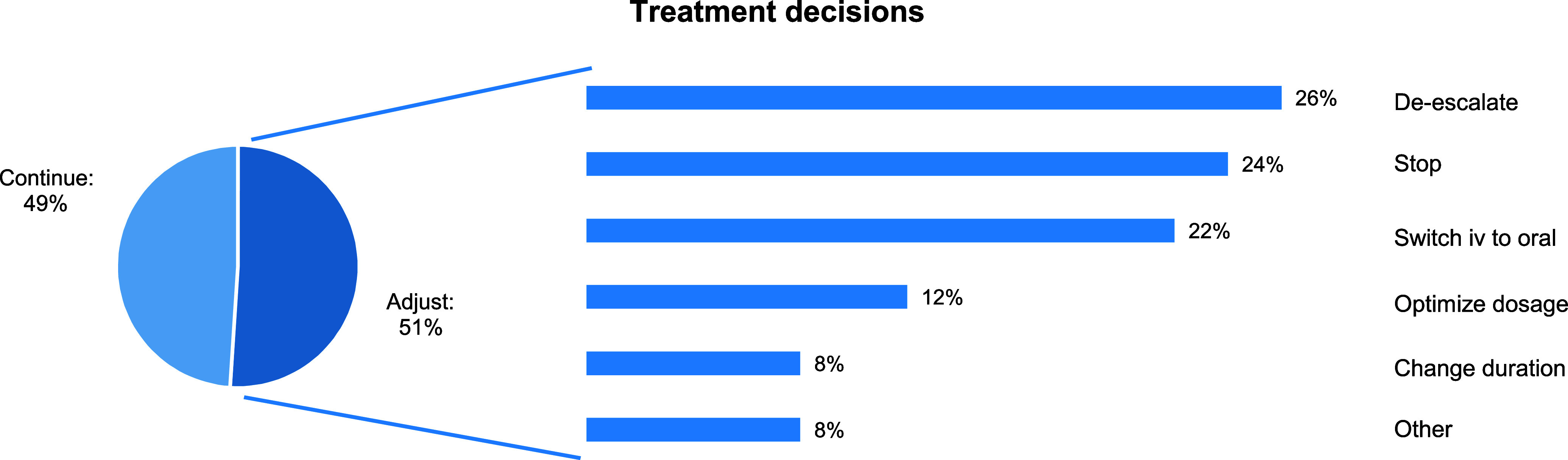
Distribution of treatment decisions made during audit and feedback by decision type. Percentages in the pie chart refer to the total number of 144 decisions made. Percentages in the bar chart refer to the total number of 74 adjustments performed.

During the case audits, 144 treatment decisions were made for 127 audited patients, as 17 patients used two different antimicrobials at the time of the audit. For 67 of the 127 patients (52.8%), the case audit resulted in one or more adjustments of therapy. The following 74 treatment modifications were agreed upon for these 67 patients: treatment de-escalation (n = 19), treatment stopped (n = 18), switching from intravenous to oral treatment (n = 16), dosage optimization (n = 9), adjustment of therapy duration (n = 6) and other interventions, such as switching to a different antimicrobial based on culture results or performing therapeutic drug monitoring (n = 6). For 70 antimicrobials used, the joint decision of the medical specialists was to continue the current treatment (see Figure 3 for the percentage distribution of the treatment decisions).

In 110 of the audited patients (86.6%), the bedside physician ordered microbiologic diagnostics prior to initiation of antimicrobial therapy. During the case audit on day 2 of treatment (partial), culture results were available for 102 patients (80.3%). Agreement between the clinical microbiologist and bedside physician on treatment was reached in all patients. Within 24 hours after the case audit, infection treatment was in line with these agreements for 136 of the decisions made, reflecting a compliance of 94%. The causes for noncompliance were patient deterioration (n = 3), a second physician who disagreed after the case audit (n = 2), early discharge (n = 1), forgetting to change treatment (n = 1), and unclear (n = 1).

During the intervention period, the daily activated HIS overview showed that one or more new patients were suitable for a case audit every 2 or 3 days. The mean time needed to select and prepare the case-audits was 38.6 minutes per day (range: 20–120 minutes; depending on the number of patients per day). Divided over the 127 audited patients, this was 24.9 minutes per patient. The time spent discussing the patient with the bedside physician was recorded for 76 case-audits and, took 5.8 minutes per patient (range: 1–15 minutes).

## Discussion

In line with the findings of other studies,^13,21^ in this study, audit and feedback provided by an AMS team for patients using antimicrobials for at least 2 days, significantly reduced both the DDD used and LOS for the audited patients. The intervention, however, did not reduce the percentage of patients using antimicrobials or the mean antimicrobial use of all patients admitted to our two urology wards. One explanation for this could be our relatively good starting position, where the quantity of antimicrobials used, as well as the number of patients using antimicrobials, was already low, decreasing the opportunities for further improvement. The reduction in antimicrobial use by the audited patients was not enough to make a difference in total antimicrobial use. Another explanation could be that the effects of audit and feedback did not transcend those of the individual audited patients. This could be caused by the fact that, at our hospital the case audits were performed by telephone. The hospital has multiple locations, which makes it impossible for the clinical microbiologist of the AMS team to discuss treatment with the bedside physician face-to-face on the ward. The educational effect of a case audit might be greater when performed face-to-face, as the positive effect of face-to-face communication on behavior is seen by others.^22–25^

Compared to the findings of Dik *et al*, where case audits were held face-to-face and the positive effects of case audits did transcend audited patients, the added value of in-person communication is underscored by two findings. First, during the phone calls in our study, the bedside physicians did not suggest additional patients for a case audit. In the study by Dik *et al*, 19 extra patients were discussed in addition to the 114 patients selected by the AMS team. Second, the time spent per case audit in our study was approximately 5 minutes, while face-to-face case audits lasted 10–15 minutes, indicating that more information was exchanged. Additionally, the percentage of therapy adjustments proposed during the case audits differed. In our study, antimicrobial treatment was adjusted for 53% of the audited patients, whereas Dik *et al* reported that 75% of patients underwent adjustments.^16^ A higher percentage not only increases the impact of the intervention but also potentially the educational effect.

The possibility of a transcending effect was an important reason for conducting this study, in search of an effective and efficient AMS strategy with a large impact. Although automatically generated information from the HIS was used as much as possible, the transfer of relevant patient information from the system, via the pharmacist, to the clinical microbiologist remained a labor-intensive activity. Overall, the audit and feedback process proved to be time-consuming, as is also seen by others.^11,26,27^ We required approximately 30 minutes for each case audit, including preparation time. Because our study revealed a reduction in antimicrobial use only in audited patients, a larger time investment would be needed to achieve a hospital-wide improvement in antimicrobial use through audit and feedback, either by conducting more face-to-face audits or by conducting a case-audit for every admitted patient using antimicrobials. These findings are confirmed by research from Campbell *et al* ^15^ but are unfeasible for many AMS teams.

Our study demonstrated that 2 days of therapy is an appropriate moment for an AMS case audit. Although it is frequently seen that AMS teams wait more than 2 days for audit and feedback, our finding is in accordance with other findings emphasizing that early interventions by AMS teams are beneficial to patients.^28,29^ The information available during the case audits, such as culture results, available in 80% of the cases from our study, infection parameters, and guideline knowledge from the AMS team, enabled physicians to sooner adjust treatments accordingly. The effectiveness of the case audits and the percentage of patients with treatment changes after the audit, namely 53%, confirm this. Furthermore, since, in our 3.5-year study period, only 42% of all urology patients using antimicrobials for therapeutic reasons, used antimicrobials for more than 2 days, prolonging the time until audit and feedback would further reduce the number of patients suitable for a case audit.

The reduction in antimicrobial use and LOS in audited patients can be explained by the large contribution of de-escalating, stopping, and switching from intravenous to oral treatment to the total number of treatment modifications. Together, these agreed-upon decisions accounted for 72% of all treatment alterations during the case audits.

There are some limitations to our study. We chose to confine our study to the urology wards of the hospital, which raises the potential question of generalizability to other wards. Furthermore, the study was executed under a nonrandomized historical cohort design; the possibility of selection bias or confounding factors contributing to changes in antimicrobial use and LOS cannot be excluded. Information on underlying disease and possible comorbidities was not available, so there may be unrecognized differences, such as differences in disease severity between patients in the intervention and the control groups. However, the baseline characteristics of the case-audit patients and the cohort patients were similar, and there were no changes in the hospital’s antimicrobial policy or patient population during the study period. Additionally, the time-series analysis effectively mitigated confounding by seasonal trends in antimicrobial use.

This study underscores the effectiveness of audit and feedback as an AMS strategy for improving the quality of care for individually audited patients, even in the absence of face-to-face contact. Furthermore, the fact that the effect of audit and feedback does not automatically transfer to patients for whom case audits were not conducted, provides us with valuable insights into factors that are important for maximizing future AMS interventions. Audit and feedback appear to be an effective but labor-intensive strategy, especially when aiming for a hospital-wide impact. These findings encourage us to further investigate AMS-team interventions to help determine the best use of limited resources for AMS programs. We will focus on promoting sustainable prescribing changes through the smart use of decision-support technology as a promising way to reduce inappropriate antimicrobial use, aiming to extend the reach of the AMS team, and to better support and equip infection-treating physicians.^30^ In addition, we will continue to conduct case audits, but more selectively for high-risk patients and high-risk antimicrobials, and face-to-face when possible.
